# Root system architecture of historical spring wheat cultivars is associated with alleles and transcripts of major functional genes

**DOI:** 10.1186/s12870-022-03937-7

**Published:** 2022-12-16

**Authors:** Saman Maqbool, Suhaib Ahmad, Zarnishal Kainat, Muhammad Ibrar Khan, Ammarah Maqbool, Muhammad Adeel Hassan, Awais Rasheed, Zhonghu He

**Affiliations:** 1 Department of Plant Sciences, Quaid-i-Azam University, Islamabad, 45320 Pakistan; 2 Institute of Crop Sciences, Chinese Academy of Agricultural Sciences (CAAS), & CIMMYT-China office, 12 Zhongguancun South Street, Beijing, 100081 China

**Keywords:** RSA, Wheat, *Rht* genes, *DRO1*, *TaMOR*, RNA-seq

## Abstract

We evaluated root system architecture (RSA) of a set of 58 historical spring wheat cultivars from Pakistan representing 105 years of selection breeding. The evaluations were carried out under control and water-limited conditions using a high-throughput phenotyping system coupled with RhizoVision Explorer software. The cultivars were classified into three groups based on release year as cultivars released pre-1965, released between 1965 and 2000, and cultivars released post-2000. Under water-limited conditions a decline in 20 out of 25 RSA component traits was observed in pre-1965 cultivars group. Whereas cultivars released after the 1965, so-called green revolution period, showed a decline in 17 traits with significant increments in root length, depth, and steep angle frequency which are important root traits for resource-uptake under water-limited conditions. Similarly, cultivars released after 2000 indicated an increase in the number of roots, depth, diameter, surface area, and steep angle frequency. The coefficient of correlation analysis showed a positive correlation between root depth and yield-related traits under water-limited conditions. We also investigated the effects of green-revolution genes (*Rht1*) and some phenology-related genes such as *DRO1*, *TaMOR*, *TaLTPs*, *TaSus-2B* on RSA and identified significant associations of these genes with important root traits. There was strong selection pressure on *DRO1* gene in cultivated wheat indicating the allele fixed in modern wheat cultivars is different from landraces. The expression of *DRO1,* and *TaMOR* were retrieved from an RNAseq experiment, and results were validated using qRT-PCR. The highest expression of *DRO1* and *TaMOR* was found in Chakwal-50, a rainfed cultivar released in 2008, and MaxiPak-65 released in 1965. We conclude that there is a positive historic change in RSA after 1965 that might be attributed to genetic factors associated with favored RSA traits. Furthermore, we suggest root depth and steep angle as promising traits to withstand water-limited environments and may have implications in selection for breeding.

**Supplementary Information:**

The online version contains supplementary material available at 10.1186/s12870-022-03937-7.

## Introduction

One of humanity’s greatest challenges in 21 century is feeding the ~ 9 billion people by 2050 with continuously decreasing natural resources [1]. Extreme weather events associated with climate change have threatened crop production patterns across the globe [2]. Drought and flooding events in response to climate extremes may increase, and it is critical to devise possible strategies to enhance crop production in the forthcoming decades [3, 4]. Wheat (*Triticum aestivum* L.) is one of the major staple food crops worldwide supplying 20% calories to the global population. Wheat is sensitive to drought and heat stress, that are critical environmental adversity affecting final crop yields [5]. Breeding for drought tolerant genotypes with agronomic and adaptive traits is crucial for increasing productivity and food security among wheat-growing communities.

Root system architecture (RSA) is important for water and nutrient uptake facilitating photosynthesis and improving grain yield. It represents a fair benchmark for manipulation under soils poor in nutrition improving crop productivity [6, 7]. RSA refers to the spatial organization of root structure that includes the root’s number, length, tip number, emergence angles, depth, width, convex hull area, and root mass center [8]. To optimize the nutrient and water uptake, understanding and selection of unique RSA traits and identification of their underlying genes are equally important as above-ground components. There are specific root architectural traits that benefit crop yield by improving soil resource acquisition. For instance, a deeper RSA can extract more water and nutrients under drought conditions relative to a wider RSA in optimal conditions. Root traits contributing to plant productivity under drought stress such as long specific root length, small fine root diameter, and high root length density have also gained significant attention [9]. In wheat, seminal roots (lateral roots originating from radicle) largely determine the root architecture at the adult plant stage. Several traits of these roots such as root growth angle, seminal root number, and length can be conveniently assessed at early growth stages [10].

Recently, a genetic framework of 38 genes underpinning RSA in cereals was reported [11]. For instance, the reduced height (*Rht*) genes responsible for the green-revolution had a significant impact on root traits and coleoptile length [12, 13]. Overexpression of *TaMOR* (*More roots* in wheat) in rice plants resulted in longer main panicle, more crown roots, a higher number of primary branches and an increased grain yield. Root depth is an important trait allowing better access to nutrients and water stored in deeper soil layers thus enhancing yield [14]. *DRO1* influences root growth angle by modulating root gravitropic response. The deep rooting allele has been found to increase grain yield under drought stress [15]. Three other major QTL namely *DRO2*, *DRO3*, and *qSOR1* have been reported to control RSA under water-deficient conditions [14]. Rambla et al. [16] reported a single plant selection (SPS) approach for introgression of root traits into elite wheat germplasm. The approach combines phenotypic selection (root angle and biomass), marker assisted selection (MAS) using KASP markers for qRDM-5B, a root biomass QTL, and speed breeding to accelerate the breeding cycles.

The main challenge in breeding programs is the lack of high-throughput phenotyping platforms for root traits that can offer a proxy for field performance. Despite that, genetic improvement in RSA is an underappreciated route to a more efficient and productive wheat crop. Evidence suggests that historical improvements in wheat productivity are linked to changes in RSA [17]. Hence, designing a root system tailored to target the edaphic environment via modification of genes underpinning favorable root traits represents an ideal breeding strategy for direct selection [4]. The development and release of new adaptable varieties with desired root systems could be a promising strategy to surmount unfavorable environmental conditions. Given the above factors, this study was conducted to investigate phenotypic variability in RSA traits of historical wheat cultivars representing the breeding progress since 1911 to 2016 in Pakistan and to identify the associations of unique RSA with phenology, yield-related traits, and allelic variations and expression of important functional genes.

## Materials and methods

### Germplasm

**Fig. 1:**
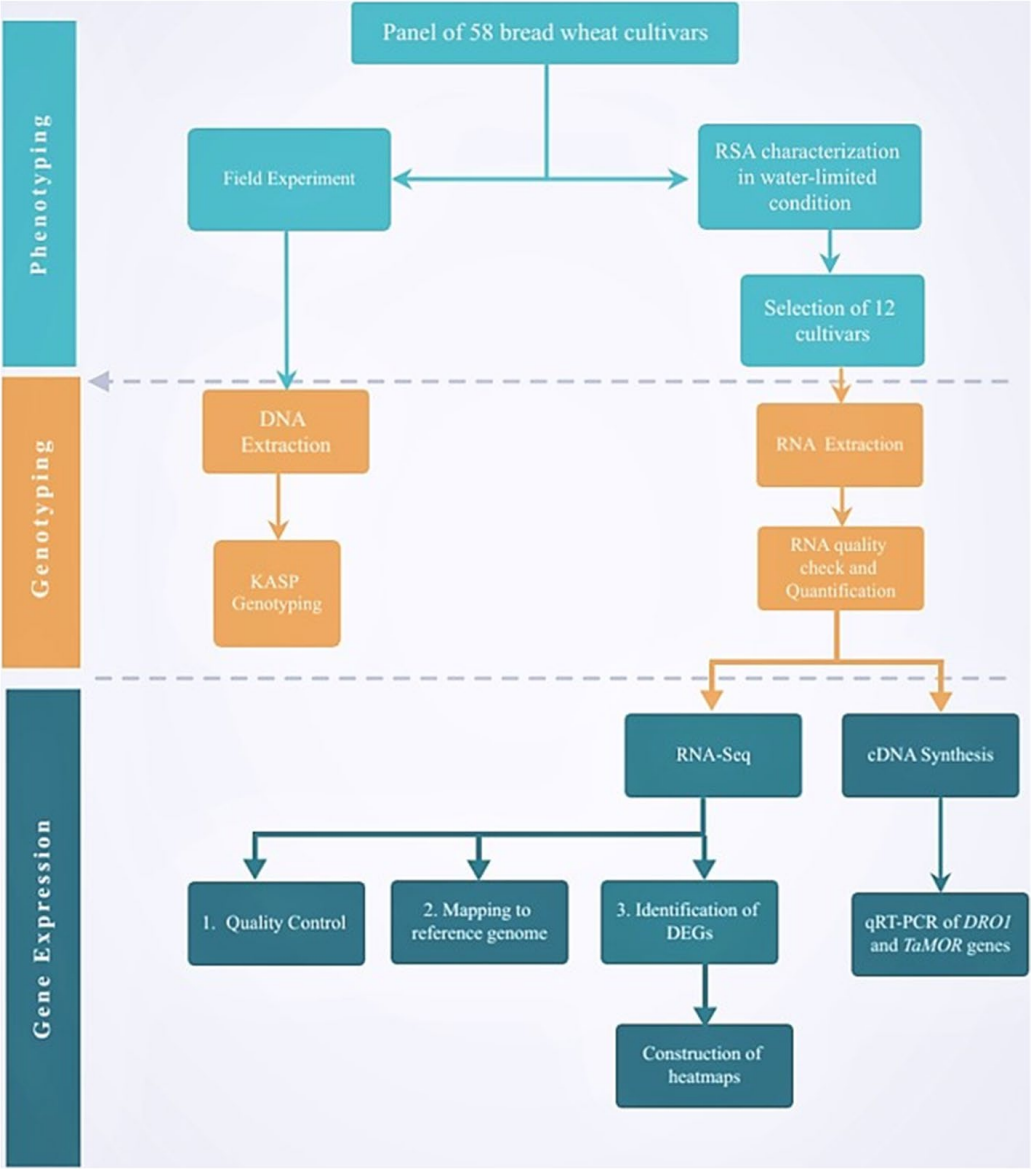
Scehmatic overview of the experimental design

A panel of 58 bread wheat cultivars released in Pakistan (1911–2016) was selected for this study. The cultivar name, year of release, and pedigree are given in Table 1. A subset of this collection was selected for RNA-sequencing and qRT-PCR validation. The schematic overview of the experiments is given in Fig. 1. All the experimentation was conducted on wheat plants, so no formal identification of plants was required, and no voucher specimen was needed to deposit in herbarium. The seeds of the cultivars used in this experiment can be accessed from National Genebank, Plant Genetic Resources Institute, National Agriculture Research Center (NARC), Islamabad, Pakistan.

**Table 1:** List of historical wheat cultivars used in the study with release year and pedigree

Cultivar	Release Year	Pedigree
T9	1911	Landrace(314818)
C-518	1933	T9/8A
C-217	1944	C516/C591
C-271	1957	C230/IP165
Dirk	1958	FORD//DUNDEE/BOBIN
Mexipak-65	1965	PJ/GB55 or PJ62/GB55
Pari-73	1973	CNO67//SN64/KLRE/3/8156
WL-711	1978	S308/CHRIS//KAL
Pak-81	1981	KVZ/BUHO//KAL/BB
Parula	1981	FKN/3/2*Frontana//Kenya 350 AD.9C.2/Gabo 55/4/Bluebird/Chanate
Barani-83	1983	BB/GLL/3/GTO/7C//BB/CNO67
Chakwal-86	1986	FORLANI/ACC//ANA
Khyber-87	1987	KVZ/TRM//PTM/ANA
Rawal-87	1987	MAYA/MON//KVZ/TRM
Inquilab-91	1991	WL 711/CROW “S”
Pasban-90	1991	INIA F66/TH.DISTICHUM//INIAF66/3/GENARO T81
Pastor	1993	PFAU/SERI-82//BOBWHITE
Bakhtawar-94	1994	AU/UP301//GLL/SX/3/PEW/4/MAI/MAYA//PEW
Parwaz-94	1995	V.5648/PARULA
Punjab-96	1996	SA42*2/4/CC/INIA//BB/3/INIA/HD832
Suleman-96	1996	F6.74/BUN//SIS/3/VEE#7
Tatara	1996	JUP/ALD’S′//KLT’S′
Chakwal-97	1997	BUC’S′/FCT’S′
MH-97	1997	NORD-DESPREZ (ND)/VG-9144//K.SONA/BLUEBIRD/3/YACO/4/VEERY-5
Auqab-2000	2000	CROW’S′/NAC//BOW’S′
Wafaq-2001	2001	OPATA/RAYON//KAUZ
AS-2002	2002	KHP/D31708//CM74A370/3/CNO79/4/RL6043/4*NAC
GA-2002	2002	DWL5023/SNB//SNB
Ufaq	2002	V.84133/V83150
Pirsabak-2004	2004	KAUZ/STAR
Pirsabak-2005	2005	MUNIA/CHTO//AMSEL
Fareed-2006	2006	PT’S′/3/TOB/LFN//BB/4/BB/HD-832-5//ON/5/G-V/ALD’S′//HPO
Seher-2006	2006	CHILL/2* STAR/4/BOW//BUC/PVN/3/2*VEE#10
Bathoor	2008	URES/JUN//KAUZ
Chakwal-50	2008	ATTILA/3/HUI/CARC//CHEN/CHTO/4/ATTILA
Faisalabad-2008	2008	PBW65/2*Pastor
Mairaj-2008	2008	SPARROW/INIA//V.7394/WL711/13/BAUS
Pirsabak-2008	2008	KAUZ/PASTOR
NARC-2009	2009	INQALAB 91*2/TUKURU
Atta-Habib	2010	INQALAB 91*2/TUKURU
Barsat-2009	2010	FRET2
AAS-2011	2011	PRL/PASTOR//2236(V6550/SUTLEH-86
Dharabi-2011	2011	HXL-7573/2*BAGULA//PASTOR
Millat-2011	2011	CHENAB2000/INQ-91
NARC-2011	2011	OASIS/SKAUZ//4*BCN/3/2*PASTOR
Punjab-2011	2011	AMSEL/ATTILA//INQ-91/PEW’S′
Galaxy-2013	2013	PUNJAB-96/V-87094//MH-97
Pakistan-2013	2013	MEX94.27.1.20/3/Sokoll//Attila/3*BCN
Pirsabak-2013	2013	CS/TH.SC//3*PVN/3/MIRLO/BUC/4/MILAN/5/TILHI
Shahkar-2013	2013	CMH84.3379/CMH78.578//MILAN
Pakhtunkhwa-2015	2015	WBLL1*2/4/YACO/PBW65/3/KAUZ*2/TRAP//KAUZ
Ujala-2016	2015	KIRITATI/4/2*WEAVER/TSC//WEAVER/3/WEAVER
Ahsan-2016	2016	Pastor/3/Altar 84/Ae. squarrosa//Opata
Borlaug-2016	2016	Sokoll/3/Pastor//HXL7573/2*BAU
Gold-2016	2016	PR-32(BAU)//INQ-91
Johar-2016	2016	KAUZ/PASTOR//V.3009
Zincol-2016	2016	OASIS/ SKAUZ//4*BCN/3/2*PASTOR/4/*T. SPELTA* PI348449/5/BACEU#1/6/ WBLL1*2/CHAPIO
Local White	–	Local White

### Phenotyping experiments

#### Evaluation of drought stress response in cigar rolls

Seeds were surface sterilized with 2% NaOCl (BioChem; active chlorine 4.0–6.0) and subsequently rinsed with distilled water three times. Ten seeds of each cultivar were placed on a Whatman quantitative ashless filter paper (Grade No.42) fixed in Petri plate and kept in dark for germination. After the emergence of coleoptiles 5 days after sowing (DAS), five uniform seedlings were transferred to germination papers rolled in cigars configuration. The cigar rolls were then placed in 1 L glass beakers totaling eight cigars in each beaker filled with 300 mL distilled water and kept as control. For drought stress treatment, 150 mL distilled water was maintained every day in each beaker [18]. Seedlings were removed 2 weeks after treatment, roots were separated and imaged using RhizoVision Crown hardware platform coupled with a machine vision camera.

RhizoVision crown hardware [19] controlled by RhizoVision Imager software was used for root image acquisition. The hardware consisted of a backlit imaging box with 65 cm × 66 cm × 91 cm dimensions that produced near binary images. The imaging box was constructed from T-slotted aluminum profiles. LED edge-lit flat panel light (65 cm × 65 cm) affixed at the back of the box provided a white background while roots gave the foreground near-black color. A root holder (22 cm × 30 cm) was constructed at the top of the box and a door handle was attached on the top of the root holder panel to assist in the placement and removal of roots. A monochrome machine vision camera (Basler acA5472–17 μm USB 3.0) with a CMOS sensor (Sony IMX183) was used to capture images. For root image analysis, an open-source software RhizoVision Explorer was used [20].

#### Field experiments

The same set of 58 wheat cultivars was evaluated for two-year (2018 and 2019) in the field at National Agriculture Research Centre (NARC), Islamabad, Pakistan using a randomized complete block design (RCBD) with two replications. The NARC site is located at 33°43′N 73°04′E and has soil electrical conductivity of 0.24dS/m. The plot size of 6 rows of 6 m length with 16 cm row-row distance was maintained. Phenotyping data for various morphological parameters including tillers per plant (TPP), plant height (PH) in cm, spike length (SL) in cm, spikelet per spike (SNPS), grains per spike (GPS), thousand grain weight (TGW), grain length (GL), grain width (GW), grain diameter (GD), and grain yield (GY) in kg per m^2^ per plot were recorded as described in our previous study [21]. The experiments were conducted under standard institutional policies and no biosafety permission was needed for this experimentation. No permission was needed to collect plants and plant materials. All procedures were conducted in accordance with the institutional guidelines.

### Genotyping for functional genes

Genomic DNA of cultivars was extracted from leaf tissues using the phenol-chloroform method. The KASP markers for genes *Rht-B1, Rht-D1,* and *TaSus2-2B,* were used from our previous work [22]. The KASP assay was prepared using 2 μl of 50 ng/μl DNA as template, 2.5 μl of 2X KASP master mix, 0.07 μl of KASP assay mix, and 2.5 μl of PCR H_2_O. The PCR was performed in 384-well formats (S1000, Thermal Cycler, USA) following conditions described by [21].

### RNAseq and qPCR-based expression of *DRO1* and *TaMOR*

A subset of 12 wheat cultivars was subjected to RNAseq to identify the genotypic variation in gene expression in seedling leaf and roots (PRJNA863398). For this purpose, seedling leaf and root tissues in triplicate were collected and subjected to total RNA extraction. Briefly, RNA was extracted using EasyPure Plant RNA Kit (ER301–01) following manufacturer instructions. RNA quality was checked on 1% Agarose gel and quantified using Nanodrop 2000 spectrophotometer (Thermo Fisher Scientific, USA). The samples were sent to Beijing Genomics Institute (BGI), China for sequencing. In summary,mRNAs were isolated and fragmented from total RNA using the oligo (dT) method for cDNA synthesis. The 50-bp single-end sequencing libraries were constructed, and sequencing was performed on BGISEQ-500 platformusing standard protocols. Adapters with unknown bases (N’s > 5%) and low quality were removed from raw reads to produce ‘clean data’ as FastQ data files using a quality control software, SOAPnuke version 2.1.6.

#### Identification of differentially expressed genes

High quality single-end reads were mapped to the bread wheat reference genome (IWGSC, INSDC Assembly GCA_900519105.1) using HISAT2 (Hierarchical Indexing for Spliced Alignment of Transcripts) software version 2.2.1 [23]. Alignment of the reference sequence with reads were performed using Bowtie [24]. Quantification of the reads was performed using featureCounts software program. Differentially expressed genes (DEGs) were identified using DeSEQ2 in R version 4.1.1. DEGs were then filtered based on their adjusted *p*-value. The threshold value for filtering was set at 0.1. To analyze the expression of *DRO1* and Ta*MOR* genes in wheat genotypes, the heatmaps were constructed using pheatmap package in R version 4.1.1.

#### Validation of DEGs using qRT-PCR

For expression analysis of *DRO1* and *TaMOR* genes*,* a subset of 12 cultivars selected based on high and least root length was germinated in cigar rolls. A total of five surface-sterilized seeds of each cultivar were placed between two germination papers and rolled in a cigar configuration. Two batches of fifteen cigar rolls totaling ten plants per genotype were placed in beakers containing Hoagland’s nutrient solution. The experiment comprised of two treatments i.e., well-watered (WW) and water-limited (WL) conditions. For well-watered treatment, 200 mL of Hoagland’s solution was added while 100 mL nutrient solution was supplied to maintain WL conditions. The nutrient solution was supplied daily to maintain a volume of 200 mL and 100 mL for WW and WL conditions respectively. The roots were harvested 15 days after germination for RNA extraction from each treatment.

#### RNA isolation, cDNA synthesis, and qRT-PCR analysis

Total RNA was extracted from a set of 12 wheat cultivars using EasyPure Plant RNA Kit (ER301–01) following manufacturer instructions. RNA quality was checked on 1% Agarose gel and quantified using Nanodrop 2000 spectrophotometer (Thermo Fisher Scientific, USA). RNA samples with 1.8–2.1 A260/A280 values were selected for cDNA synthesis. Genomic DNA was removed, and cDNA was synthesized using ABClonal ABScript III RT Master Mix with gDNA remover. The reverse transcription reaction system consisted of 4 μl 5X ABScript III RT Mix, 1 μl 20X gDNA remover mix, 1 pg-1 μg of total RNA, and 13 μl nuclease-free water making up to 20 μl. The conditions for the reaction were 37 °C for 2 minutes, 55 °C for 15 minutes, 85 °C for 5 minutes, and 4 °C to hold. The products were quantified using Nanodrop 2000 spectrophotometer (Thermo Fisher Scientific, USA) and stored at − 20 °C for subsequent qRT-PCR reaction.

The coding sequences of *DRO1* and *TaMOR* were retrieved from NCBI and were used to design common primers for all three homoeologues using Primer-BLAST (https://www.ncbi.nlm.nih.gov/tools/primer-blast). A previously designed primer pair for *TaActin* was used as an internal control. The sequences of primers used are given in Table 2. The transcription levels of *DRO1* and *TaMOR* were quantified using Livak method in a CFX384 Real-Time detection system (Bio-Rad). The reaction components were as follows: 10 μl 2X Universal SYBR Green Fast qPCR, 1 μl cDNA product (40 ng/ μl), 0.4 μl forward primer (10 μM), 0.4 μl reverse primer (10 μM) and 8.2 μl nuclease-free water to make a final volume of 20 μl. The two-step reaction conditions were as follows: 1 cycle at 95 °C for 3 minutes and cycles at 95 °C for 5 seconds, 60 °C for 30 seconds. The amplification and melting curves were confirmed after the reaction and then a standard curve for quantitative analysis was made.

**Table 2:** Sequence of primers used in qRT-PCR

Primer Name	Sequence (5′- > 3′)	Tm (°C)	Size (bp)
qRT_DRO1_F2	GACGAGTTCAGCGATTGGC	59	86
qRT_DRO1_R2	TCCTGCACTTGTGCTACCTC	59	
qRT_TaMOR_AF2	CCTACTTCTGCCACGAGCA	59	81
qRT_TaMOR_AR2	GGAGCTTGGAGACGTTGCTG	61	
TaActin_F	GGAGAAGCTCGCTTACGTG	60	140
TaActin_R	GGGCACCTGAACCTTTCTGA	60	

### Statistical analysis

Data were analyzed using a generalized linear mixed model. Mean comparisons were made with Tukey’s test at a *p* = 0.05 significance level. All statistical analyses were performed using Jamovi version 1.8. PCA analysis of root and yield related traits was performed using two principal components in Jamovi version 1.8. Correlations among root and yield traits were determined using Pearson correlation coefficient using *GGally* package in R 4.1.1. The student’s t-test was used to investigate the significant association of a marker with the target trait at a threshold probability *p* < 0.05 [25].

## Results

### Trends and genotypic differences in root traits over time

Wheat RSA traits were phenotyped in the Rhizovision Hardware Crown platform using the Cigar roll method. The set of fifty-eight wheat cultivars was divided into three groups based on breeding eras as pre-1965 (*n* = 6), 1965–2000 (*n* = 19), and post-2000 (*n* = 33). Descriptive statistics of each group with mean and range of all root traits under controlled and water-limited condition is given in Table 3. The highest depth was recorded in post-2000 cultivars with a 1.7-fold variation under water-limited conditions. A 1.3–1.35-fold variation under control and 1.5–1.52-fold variation under water-limited condition was observed in 1965–2000 and post-2000 group, respectively. For TRL, a similar trend was observed under control, however, highest mean (493) was found in 1965–2000 cultivars under water-limited conditions. A 2.2-fold variation was observed under control whereas under water-limited condition, a 2.9-fold variation was observed. For root volume, a 6.4-fold variation was observed under water-limited conditions with 1.9-, 2.5- and 2.7-fold variation in cultivar groups pre-1965, 1965–2000 and post-2000, respectively.

**Table 3:** Descriptive statistics of root and yield-related traits in fifty-eight wheat cultivars evaluated under optimum and water-limited condition

Group	Mean						Range					
	Pre-1965		1965–2000		Post-2000		Pre-1965		1965–2000		Post-2000	
Treatment	Control	Drought	Control	Drought	Control	Drought	Control	Drought	Control	Drought	Control	Drought
**MNR**	2.31	2.44	2.42	2.37	2.34	2.38	2–2.67	1.67–3	1.67–3.33	1.67–3	1.67–3.67	1.33–4.33
**MaxNR**	6.94	6.22	6.61	6.04	6.6	5.89	5.33–10	4.33–7	5–8.33	4.67–8	4.67–8.33	4.33–8.67
**NRT**	14.2	9.05	14	8.93	15.4	8.81	6.67–20	7.33–12	8.33–22.3	5.33–13.7	7.33–28.3	4.67–15.7
**TRL**	444	431	484	493	495	485	397–495	352–561	373–642	323–651	293–641	252–726
**D**	45.1	40.3	45.8	46.5	47.2	48.1	39.3–49.5	30.9–51	35–54.2	32.3–62.6	35.3–59.3	35.3–59.7
**MaxW**	169	163	184	175	188	178	150–191	135–193	147–203	137–203	150–203	120–203
**WDR**	3.9	4.33	4.12	3.99	4.15	3.96	3.1–4.72	3.14–5.6	2.92–4.96	2.91–7.15	2.77–6.93	2.86–6.09
**NtA**	360	304	365	350	385	358	331–420	199–395	299–463	212–455	235–573	230–546
**CA**	3052	2536	3217	3251	3340	3334	2587–3471	1348–3567	1983–4428	1797–5254	1828–5385	1575–5428
**S**	0.123	0.132	0.124	0.117	0.131	0.122	0.1–0.18	0.1–0.18	0.09–0.18	0.08–0.16	0.08–0.2	0.08–0.2
**LRA**	64.5	59.5	70.9	49.7	61.9	56.4	42.3–84.8	16.1–206	34.4–248	16.2–93.2	13.4–115	17.4–137
**AD**	0.988	0.852	0.906	0.867	0.932	0.899	0.92–1.15	0.68–0.98	0.76–1.02	0.71–1.15	0.78–1.1	0.72–1.16
**MD**	0.765	0.66	0.727	0.641	0.761	0.669	0.65–0.94	0.55–0.78	0.59–0.86	0.55–0.83	0.64–1.02	0.58–0.83
**MaxD**	6.19	5.21	5.27	6.25	5.55	5.74	4.89–7.01	3.98–6.67	2.97–7.74	3.73–9.41	3.42–7.4	2.94–9.73
**P**	824	818	909	936	927	917	737–908	669–1067	685–1210	612–1237	556–1204	472–1364
**V**	612	384	471	522	518	515	500–720	195–583	284–654	256–999	281–863	279–1247
**SA**	1384	1134	1358	1322	1435	1344	1262–1567	737–1493	1093–1702	806–1718	909–2142	918–1996
**ARO**	18.7	17	18	17	17.9	17.9	16.2–23.1	13.5–23.6	12.8–22.4	13–21.4	13.8–23.5	12.3–23.8
**StAF**	0.857	0.865	0.853	0.872	0.858	0.862	0.78–0.89	0.72–0.93	0.78–0.92	0.8–0.94	0.77–0.93	0.74–0.93
**MAF**	0.08	0.0933	0.0905	0.0858	0.0845	0.0934	0.06–0.14	0.04–0.22	0.05–0.15	0.04–0.15	0.04–0.16	0.05–0.19
**SAF**	0.06	0.045	0.0584	0.0421	0.0567	0.0444	0.03–0.09	0.03–0.06	0.02–0.1	0.02–0.06	0.03–0.1	0.02–0.08
**RLD**	423	414	467	473	477	464	369–475	335–545	354–624	311–633	277–617	217–696
**PAD**	353	304	373	342	392	353	326–437	202–392	291–483	211–463	230–607	181–546
**SAD**	1110	955	1171	1076	1230	1110	1025–1373	633–1231	915–1516	664–1455	722–1907	569–1716
**VD**	277	213	277	234	299	252	233–360	115–271	206–386	130–364	168–563	135–416

*MNR* Median number of roots, *MaxNR* Maximum number of roots, *NRT* Number of root tips, *TRL* Total root length.mm, *D* Depth.mm, *MaxW* Maximum width, *WDR* Width-to-Depth ratio, *NtA* Network area. mm^2^, *CA* Convex area.mm^2^, *S* Solidity, *LRA* Lower root area.mm, *AD* Average diameter.mm^2^, *MD* Median diameter, *MaxD* Maximum diameter.mm, *P* Perimeter.mm, *V* Volume.mm^3^, *SA* Surface area.mm, *ARO* Average root orientation. Deg, *SAF* Shallow angle frequency, *MAF* Median angle frequency, *StAF* Steep angle frequency, *RLD* Root length diameter.mm, *PAD* Projected area diameter.mm, *SAD* Surface area diameter.mm, *VD* Volume diameter.mm

Significant variations (*P* < 0.05) among genotypes were found for all root traits except maximum diameter (MaxD) and width to depth ratio (WDR). The mean squares of each trait are given in Table 4. For treatment and variety x treatment interaction, significant differences were also found in 12 and 14 root traits, respectively.

**Table 4:** Analysis of Variance (ANOVA) for root traits of wheat cultivars analyzed under water-limited conditions

Source	Treatment	Replication	Variety	Treatment*Variety
df	1	2	57	56
Traits				
Median Number of Roots	0.018 ns	0.32 ns	1.05***	0.51 ns
Maximum Number of Roots	3.68***	39.50 ns	2.22**	2.35 ns
Number of Root tips	3074.9***	11.4 ns	66.1***	30.8 ns
Total Root Length (mm)	3124 ns	4768 ns	32530***	12542**
Depth (mm)	5.84 ns	36.76 ns	134.00**	82.54 ns
Maximum Width (mm)	7610***	3266***	1440***	597*
Width to Depth Ratio	1.20 ns	1.96 ns	1.85 ns	1.38 ns
Network Area (mm^2^)	62924***	25774***	18779***	6953***
Convex Area (mm^2^)	113,046 ns	841,493 ns	2.66e+ 6***	1.33e+ 6 ns
Solidity	0.0038 ns	0.0046 ns	0.003***	0.0015 ns
Lower Root Area (mm^2^)	9755*	1720 ns	3499*	3613*
Average Diameter (mm)	0.18**	0.07*	0.03***	0.02*
Median Diameter (mm)	0.073***	0.09***	0.02**	0.01 ns
Maximum Diameter (mm)	11.09 ns	2.43 ns	3.73 ns	5.81**
Perimeter (mm)	1.93 ns	15,388.28 ns	113,654.24***	44,774.66*
Volume (mm^3^)	3463 ns	113,688 ns	69869*	92674***
Surface Area (mm^2^)	741225***	318456**	252319***	120016***
Average Root Orientation (deg)	22.45 ns	4.72 ns	25.36***	14.65 ns
Shallow Angle Frequency	0.008 ns	0.004 ns	0.008***	0.004 ns
Medium Angle Frequency	0.002 ns	0.004 ns	0.004***	0.002 ns
Steep Angle Frequency	0.02***	4.94e-5 ns	9.68e-4*	9.16e-4*
Root Length Diameter (mm)	5277 ns	3868 ns	32154***	12321*
Projected Area Diameter (mm)	127581***	25098**	21751***	7110**
Surface Area Diameter (mm)	1.26e+ 6***	247701**	214674***	70178**
Volume Diameter (mm)	206211***	40737***	17647***	5774**

* Significant (*P* < 0.05); ** Significant (*P* < 0.01); *** Significant (*P* < 0.001); ns Non-significant (*P* > 0.05); df: degree of freedom; All the values are mean squares

### Pearson coefficient of correlation and principal component analysis

**Fig. 2:**
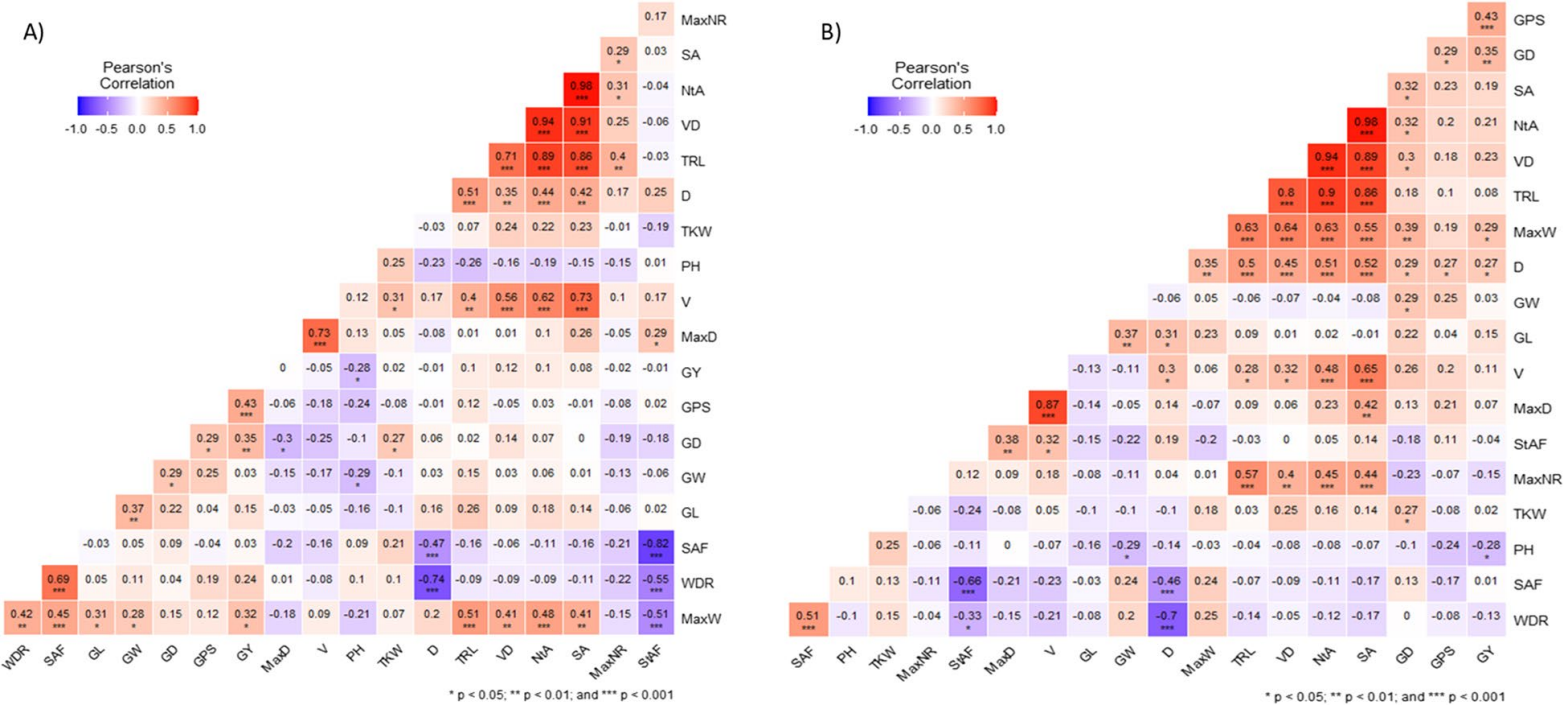
Pearson’s correlation coefficients describing association of root and yield traits of 58 historical wheat cultivars **A**) control **B**) water-limited condition; * Significant (*P* < 0.05); ** Significant (*P* < 0.01); *** Significant (*P* < 0.001). The trait abbreviations are mentioned in Table 2

The coefficient of correlations between various root traits are illustrated in Fig. 2. Significant (*P* < 0.05) positive correlations were found among various root and yield-related traits. Under water-limited conditions, depth (D) showed significant positive correlation with volume (V), grain length (GL), maximum weight (MaxW), total root length (TRL) and volume diameter (VD), whereas a strong negative correlation was found with WDR and shallow angle frequency (SAF). TRL had significant positive correlations to V, D, MaxW, maximum number of roots (MaxNR), surface area (SA), and network area (NtA). Among yield-related traits, only grain density (GD) was found positively correlated to root traits such as D, MaxW, NtA, and SA.

Under control condition, steep angle frequency (StAF) had strong negative correlations to WDR, SAF, and MaxW, whereas D was negatively correlated to WDR and SAF. MaxD also exhibited a negative correlation to GD. MaxW had significant positive correlations to various root traits (SA, NtA, VD, TRL, SAF, WDR) and yield-related traits (grain yield (GY), grain weight (GW), GL). Similarly, V showed a significant positive correlation to thousand kernel weight (TKW), SA, NtA, VD, and TRL. A strong positive correlation was also found between D and TRL, SA, VD, and NtA. The TRL was also significantly positively correlated to MaxNR, SA, NtA and VD.

**Fig. 3:**
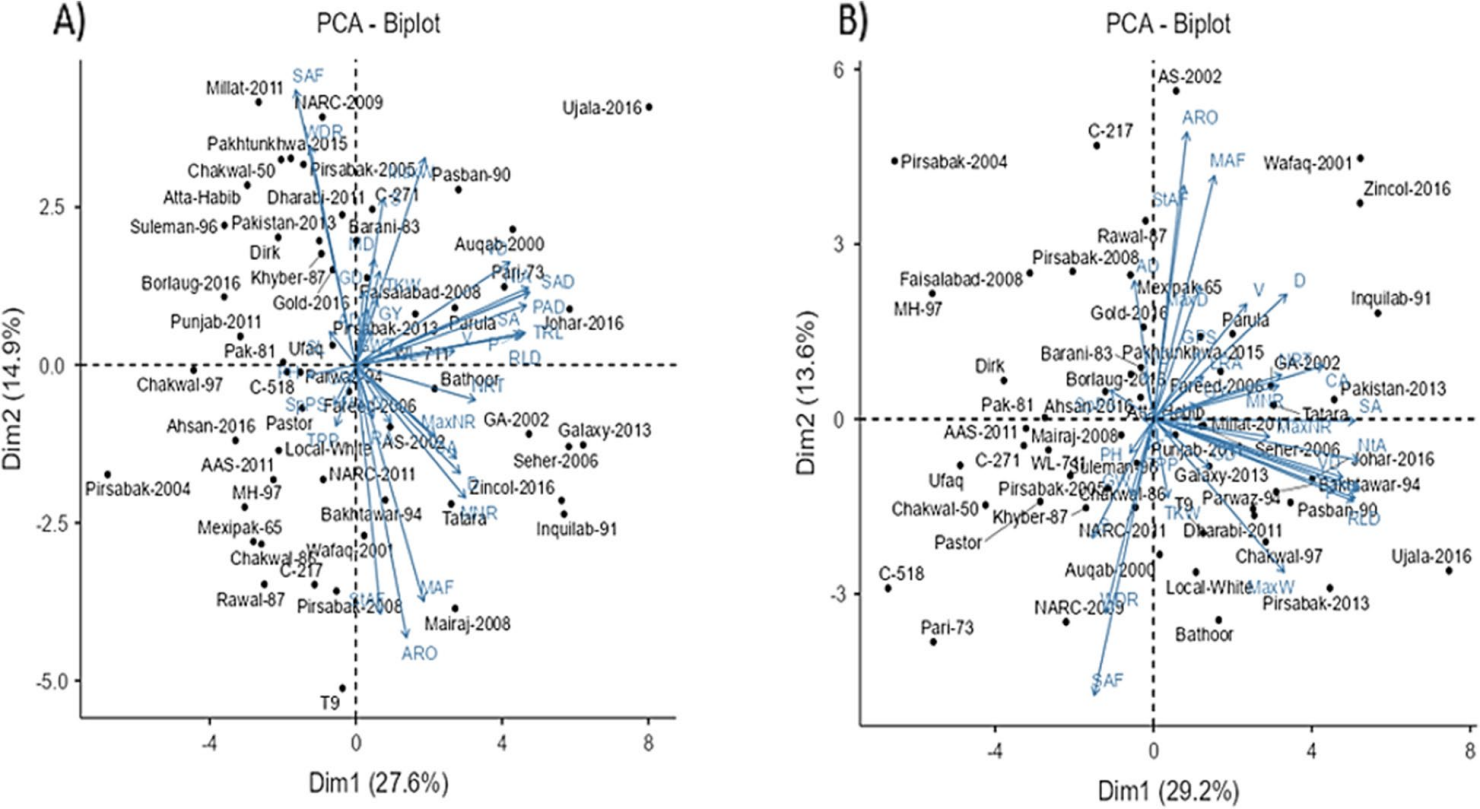
PCA biplot showing trait vectors and position of genotypes tested under **A**) control and **b**) water-limited conditions. The trait abbreviations are mentioned in Table 2

The PCA biplots exhibiting relationships among genotypes and various root and yield-related traits are illustrated in Fig. 3. The first two axes together explained 42.5% variation in control and 42.8% variation under drought stress. The angle of the vectors indicated marked differences among the association of various root traits with yield parameters in control and drought conditions. The SAF and WDR indicated remarked differences under control and water-limited conditions (Fig. 3A, B).

### Allelic variations for functional genes and their association with root traits

The KASP markers were used to identify the allelic variations for 19 functional genes. Allelic effects and frequencies of some important genes are given in Table 5. Among *Rht1* genes, *Rht-D1a* had the highest frequency (89.6%). Among *TaSus2-2B*, *TaMOC*, and *TaLTPs,* Hap-H, A, GC alleles were found dominant with 100% allelic frequency in pre-1965 cultivars. In the other two breeding groups (1965–2000 and post-2000), Hap-L (94.7, 96.9%), A (63.1, 66.6%), and GC (100% in both groups) were found dominant.

**Table 5:** Phenotypic effects and frequencies of important functional genes in historical spring wheat cultivars of Pakistan

Gene	Alleles	Frequency (%)				Allelic Effect
		Pre-1965	1965–2000	Post-2000	Overall	
*Rht-B1*	*Rht-B1a*	83.3	10	21.2	27.6	Wild type
	*Rht-B1b*	16.6	85	78.8	72.4	Semi-dwarf
*Rht-D1*	*Rht-D1a*	100	85	12.1	89.6	Wild type
	*Rht-D1b*	0	10	87.9	10.3	Semi-dwarf
*Rht1* haplotypes	*Rht-B1a/Rht-D1a*	100	15.8	9.0	19.0	Wild type
	*Rht-B1b/Rht-D1a*	0	73.7	78.8	70.7	Dwarf/Wild type
	*Rht-B1a/Rht-D1b*	0	10.5	12.1	10.3	Wild type/Dwarf
*TaSus2-2B*	Hap-L	0	94.7	96.9	86.2	Low TGW
	Hap-H	100	5.3	3.03	13.8	High TGW
*TaMOC*	Hap-A	100	63.1	66.6	69.0	Low grain number
	Hap-G	0	36.8	33.3	31.0	High grain number
*TaLTPs*	Hap-GC	66.6	100	100	96.55	Plant height
	Hap-GT	33.3	0	0	3.44	

**Fig. 4:**
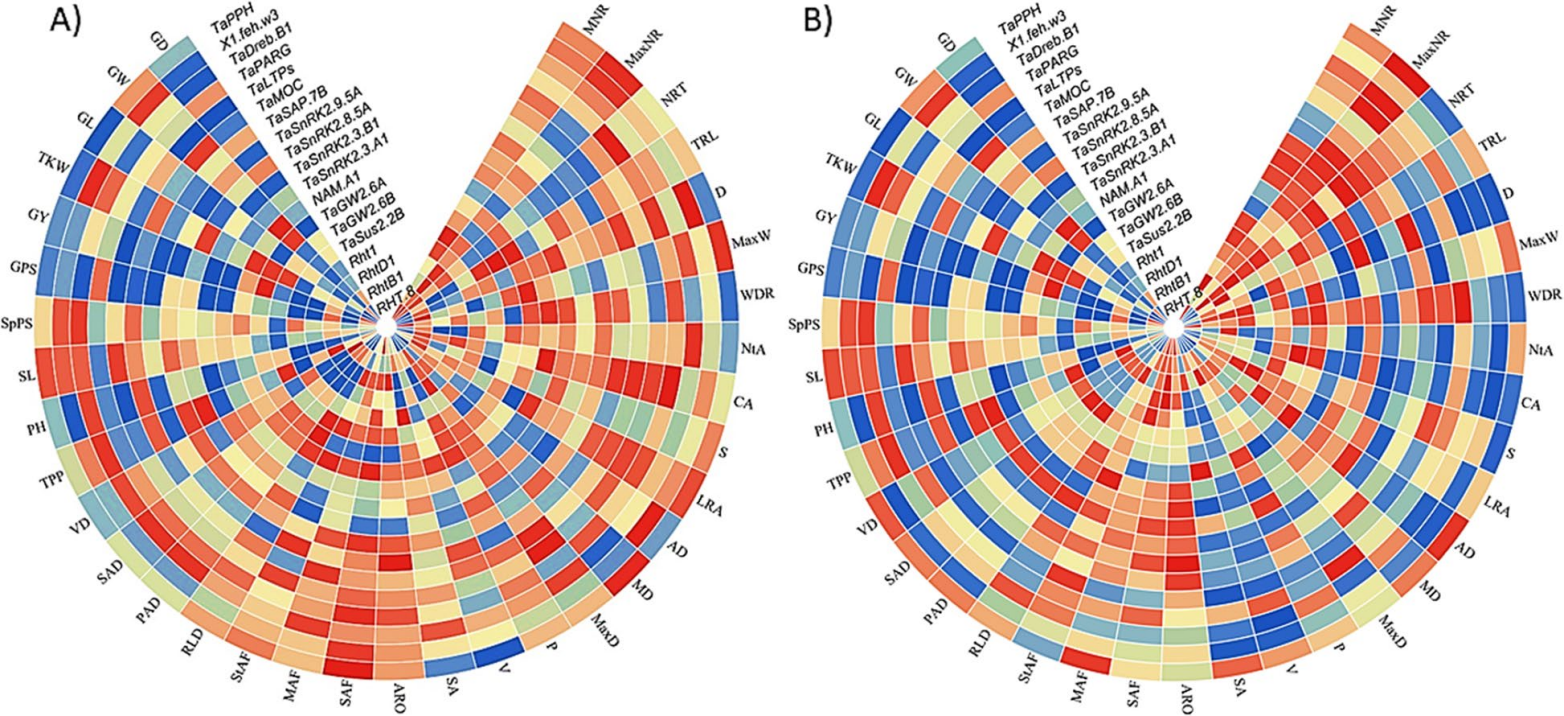
Heat maps showing significant allelic effects of functional genes on important root traits. **A**) control **B**) water-limited conditiion. The legends for *p*-value is shown on the upper right corner. The trait abbreviations are mentioned in Table 2

The associations of the alleles with root traits demonstrated remarked differences (Fig. 4). *Rht-B1* and *TaMOC* had no significant effect on any root trait tested. The *TaSus2-2B*, *TaSnRK2.3-B1*, *TaSnRK2.9-5A*, *TaDreb-B1*, *1fehw3*, and *TaPPH* were significantly associated with root depth under water-limited conditions. *TaDreb-B1* and *1fehw3* also had significant association with root volume. The *TaSnRK2.3-B1* and *TaLTPs* were significantly associated MaxW. None of the alleles were found associated with root traits related to number and diameter. Individual *Rht-B1* and *Rht-D1* did not show any association with any traits except when combined in haplotypes. *Rht1* haplotypes had significant effects on TRL, MaxW, NtA, SA, VD, convex area (CA), average diameter (AD), perimeter (P) projected area diameter (PAD), and surface area diameter (SAD) under control. No significant association of any allele was found with D under control condition.

### Analysis of genetic variations at major root biomass QTL qRDM-5B

**Fig. 5:**
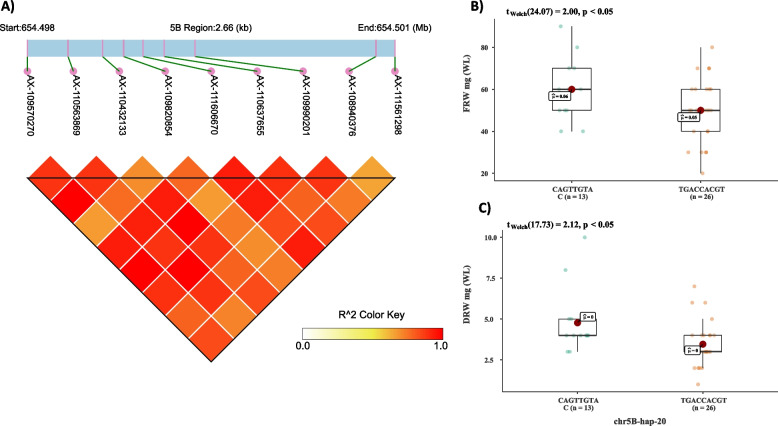
A linkage disequilibrium (LD) based heatmap showing LD (r^2^) values between 9 SNP markers on chr5B-hap-20 (**A**), and box-plot showing association of two haplotype alleles of chr5B-hap-20 with fresh root weight under control and drought stress (**B**), and dry root weight under control and drought stress (**C**)

A major QTL, qRDM-5B, for dry root biomass was identified on chr5B [26]. Single plant selection protocol including the haplotype of this QTL was suggested to be used for selection of desired RSA in wheat [16]. Since the 660 K data was available for this collection, all the SNPs within the 653.8 to 654.9 Mb were retrieved and haplotypes were constructed based on linkage disequilibrium. In total, 21 different haplotypes were identified. Out of these, chr5B-hap20 consisted of 9 SNPs and four different haplotype alleles (Fig. 5a). The frequency of two alleles was very rare and were present only in two cultivars and were excluded from the association analysis. Two haplotype alleles of chr5B-hap-20 viz. CAGTTGTA (*n* = 13) and TGACCACGT (*n* = 26) were used for the association analysis. The student’s t-test was significant where the former haplotype allele was associated with higher root dry weight (Fig. 5b), and the latter haplotype allele was associated with lower root dry weight (Fig. 5c).

### Nucleotide variations and gene expression analysis of *DRO1* and *TaMOR*

**Fig. 6:**
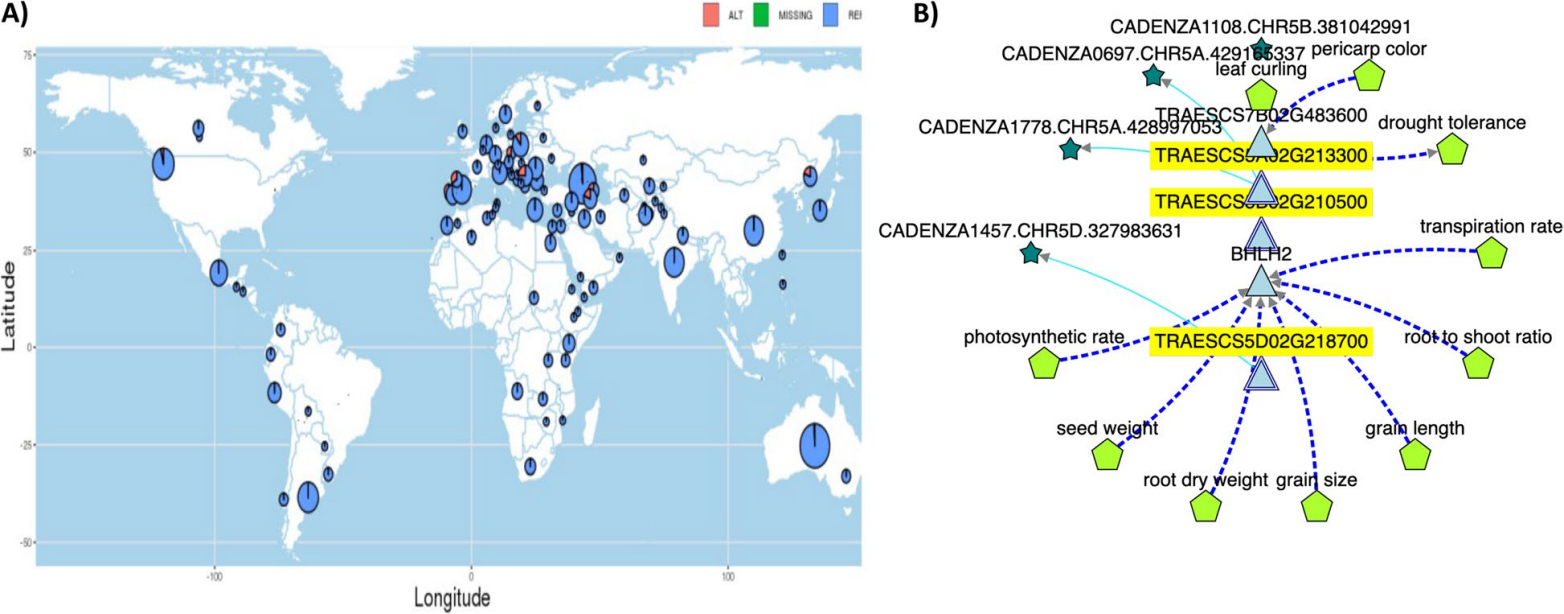
**A** Geographic hapmap of *DRO1* showing frequency of SNP on 428,996,691 bp on chr5A within *DRO1* gene, **B** The KnetMiner network of *DRO1* illustrating associated traits, transcription factors and SNPs within Cadenza TILLING population

*DRO1* and *TaMOR* genes were selected for expression analysis in roots. A 660 K SNP array data on this cultivars collection was used to extract SNPs within *DRO1* and *TaMOR* genes (Table 6). In *DRO1*, a total of 6 SNPs were identified out of which AX-109484887, and AX-108936574 caused missense mutation on *DRO1-5B*. Since no missense mutation was identified in *DRO-5A*, we then used SnpHub portal to identify any missense mutation in global wheat collection based on exome capture data. A SNP was identified with a very rare missense mutation in global wheat collection, and geographic map of *DRO-A1* haplotypes is shown as Fig. 6a. Knetminer gene network of *DRO1* showing associated traits and SNPs in Cadenza TILLIG population is shown (Fig. 6b).

**Table 6:** List of SNPs within *DRO1* and *TaMOR* genes extracted from 660 K SNP array data

Gene	ID	SNP	Description	Position	Mutation effect	Amino acid change
*DRO1-5A*	TraesCS5A02G213300	AX-95176950	G - > A	428,996,432	synonymous variant	p.Arg183Arg
*DRO1-5B*	TraesCS5B02G210500	AX-110437936	G - > A	381,042,058	Downstream gene variant	
		AX-109484887	G - > C	381,042,744	Missense variant	p.Pro165Arg
		AX-108936574	T - > C	381,042,958	Missense variant	p.Met94Val
		AX-110640724	C - > A	381,044,338	Intron variant	
*DRO1-5D*	TraesCS5D02G218700	AX-95176950	C - > T	327,632,470	Synonymous variant	p.Arg87Arg
*TaMOR-4A*	TraesCS4A02G415400	AX-108991022	C - > A	685,380,738	Missense variant	p.Lys287Asn
*TaMOR-4B*	TraesCS4B02G316200	AX-110480473	G - > C	605,692,196	Downstream gene variant	
*TaMOR-4D*	TraesCS4D02G312800	AX-108991022	A - > C	478,998,487	missense variant	p.Asn284Lys

Since all the SNPs were fixed in the 58 cultivars panel with no allelic variation, therefore 660 K SNP array data was retrieved for 159 cultivars and landraces from Pakistan for in-depth analysis of SNP states. It was revealed that all the SNPs in *DRO1* were fixed in wheat cultivars (113 out of 159) and alternate allele was identified in landraces (46 out of 159), and some older cultivars like Chenab-70, Local White and Kharchia. While AX-110640724 was completely fixed in all 159 accessions with no variation. Four SNPs were identified in *TaMOR*, of which AX-108991022 was present on both A- and D- homeologue caused missense variations, and only three landraces had the nucleotide change.

**Fig. 7:**
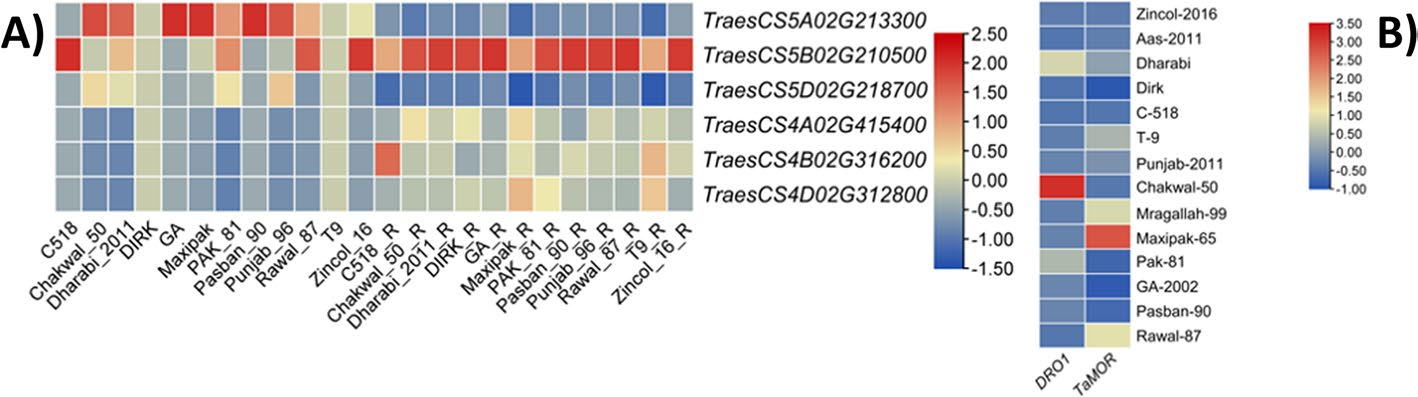
Heat maps exhibiting expression of *DRO1* and *TaMOR* genes in 12 wheat cultivars **A**) RNA-seq based expression in leaves and roots; 1–12 leaf, 13–24 roots **B**) qRT-PCR based normalized expression ratio in roots across three homoeologues based on consensus PCR markers within three homoeologues

Transcriptome analysis of twelve wheat genotypes showed significant differences in expression patterns of genes (Fig. 7a). *DRO1* was expressed in both leaf and roots with variations in leaf tissues among genotypes. In roots, the highest expression of *DRO1* in all genotypes was found in B homoeologue (TraesCS5B02G210500) with negative expression in A (TraesCS5A02G213300) and D homoeologues (TraesCS5D02G218700) relative to B homoeologue. *TaMOR* exhibited no expression in leaf tissues in all genotypes, hence, differentially expressed in root tissues. The highest expression in A homoeologue (TraesCS4A02G415400) was found in Chakwal-50 cultivar with 0.70 tpm value whereas in B homoeologue (TraesCS4B02G316200) was found in C-518 (0.88 tpm) following Zincol-16 (0.45 tpm). The highest expression in D homoeologues (TraesCS4D02G312800) was found in Pak-81 (1.10 tpm) following GA-2002 (0.54 tpm). qRT-PCR for *DRO1* expression (Fig. 7b) indicated the highest normalized expression ratio in Chakwal-50 (29.45) whereas the lowest expression was observed in Dirk with an expression ratio of 0.18. MaxiPak-65 exhibited the highest expression of *TaMOR* (4.01) while the lowest expression was recorded in Dirk with a 0.06 expression ratio.

## Discussion

In this study, we evaluated a set of historical wheat cultivars representing the diversity of Pakistan for changes in RSA over time. Root architectural traits such as lateral root number, volume, root length, density, and surface area aid in water uptake from water-deficient soils [27]. The root system of each of three breeding groups responded differently to water-limited conditions. Pre-1965 cultivars exhibited a decrease in 20 root traits under drought stress as compared to control conditions including length, density, volume, and area related root traits. Whereas cultivars released between 1965 and 2000 showed a positive response to drought in terms of length, volume, and diameter but the number of roots was decreased. The number of roots was increased in post-2000 cultivars under water-limited conditions along with root surface area and diameter. Zhu et al. [10] analyzed modern high yielding cultivars of northwestern China and showed that primary seminal root length was positively correlated with yield. Roots of higher-yielding modern varieties were simpler,compact and grew deeper than low-yielding modern varieties. Their results were also consistent to this study that the success of wheat breeding for higher yields over past 100 years in northwestern China has been in part due to unconscious group selection on root traits, resulting in smaller, less branched, and deeper roots, suggesting a direction for further increases in crop yield in the future. However, to deliver increases in crop yields, context-dependent optimization of root systems is crucial. For instance, the first cloned gene *DRO1* controlling narrow root angle in rice was described to provide yield benefit under limited water conditions [26]. McGrail and McNear [28] also concluded while studying RSA of cultivars representing two centuries of breeding progress that landraces and modern cultivars have contrastingly different RSA, while RSA of intermediate and modern wheat cultivars did not vary significantly.

The response of root growth to water deprivation usually includes growth enhancement of first- and second-order roots and inhibition of lateral roots growth. When water scarcity is severe, a drought avoidance program is implemented to direct root growth and branching into resource-rich regions [29]. Root biomass in wheat is a multi-trait function including number, length, and diameter of seminal and nodal roots the response of which to drought might be positive, negative, or no response. In our study, the positive response of some important RSA traits of post-1965 cultivars to breeding for drought stress tolerance improved the cultivars ability to transport more assimilates to roots for an efficient root system required for resource uptake. In line with our findings, Ephdaie et al. reported that under severe drought intensity (36%), wheat cv. Pavon76 used a large portion of plant-available water to increase its root biomass thus leaving behind a small amount insufficient for grain filling causing a reduction in grain yield [30]. It has been well established while comparing landraces and modern cultivars that total root biomass significantly differed between Turkish landraces and modern cultivars [31], old and modern Mediterranean wheat [32], old and modern American wheat [33], drought tolerant landraces and CIMMYT-derived wheat [34], and drought tolerant landraces and modern American wheat [31]. All these studies have consensus that landraces or older cultivars have greater biomass compared to modern cultivars.

Root angle is also an important drought-adaptive trait that directs the horizontal and vertical distribution of roots into the soil. A strong link between steep root angle and deep rooting has been reported in wheat [35]. The narrow, compact and deep-rooted architecture of higher-yielding cultivars appears to minimize water use early in the season and subsequently enhance access to water alter in the developmental stages [10]. It has also been reported that introduction of *DRO1* into a shallow rooting rice cultivar enabled the cultivar to increase deep-rooting and yield under drought stress conditions [15]. Under water-limited conditions, there was a strong correlation of root depth to other favourable root traits. For instance, root depth was positively correlated to StAF, GD, GL, GY, GPS under drought stress. Zhu et al. [10] also identified negative correlation between seminal root growth angle and grain yield in modern wheat cultivars, which indicated deeper roots likely favored resource acquisition for enhancing grain yield. We also found a positive correlation between depth and StAF in both well-watered and water-limited conditions. Under drought stress, pre-1965 cultivars exhibited a decrease in root depth contrary to other breeding groups after 1965. Progressive enhancement of rooting depth in 1965–2000 and post-2000 cultivars indicates enhanced tolerance to drought stress. Hermanska [36] reported a highly significant correlation between root system size and grain yield. Therefore, the enhanced grain yield in post-2000 cultivars might be attributed to improved root systems tailored to extract water and nutrients from deep soil layers under water-limited conditions. Root growth angle increases towards gravity due to greater expression of *DRO1.* This gene enabled the scientists to cope with the drought problems by allowing the roots to penetrate deeper thus aiding in yield enhancement even under limited water supply [14]. In our gene expression studies, we found a strong expression of the *DRO1* gene in all cultivars tested that supports our findings of increased depth under drought stress as compared to control in cultivars released after 1965.

Although extensive efforts have been made to identify the association of functional genes with the above-ground phenological traits in wheat, and functional markers have been used by various groups to tag favorable alleles in germplasm [22]. Although various Genome-wide association studies are available for genome-wide association of SNP markers with RSA. However, less efforts have been made to identify the useful allelic variation with RSA, to further use such information for breeding desirable RSA. RSA may hold the key for the “second green revolution” therefore the effect of semi-dwarfing alleles (*Rht*) on the root system is of prime importance [37]. Generally, the effects of *Rht1* alleles on root systems are less clear, with studies in different growing conditions producing contradictory results. We found a significant association of reduced height alleles (*Rht*) with some root traits. *Rht8* allele is linked to reduced plant height and the highest frequency (90.9%) was recorded in post-2000 cultivars. The results are consistent with our field data where the least plant height was recorded in post-2000 cultivars. The gibberellin (GA-3) insensitive green revolution allele *Rht-D1b* linked to dwarfism was also frequent (87.8%) in post-2000 cultivars. Similar to our findings, Hurd [38] reported that semi-dwarfing wheat lines had larger root systems as compared to tall control. Whereas contradictory to Laperche et al. [39], we found no significant impact of *Rht-B1* on root architecture that might be attributed to differing growing conditions [40].

The low heritability of RSA components traits a major challenge because most of the traits are controlled by many genes with minor effects [41]. Very few QTL with large effect are known for RSA in wheat, of which a QTL on chr5B, qRDM-5B, is prominent and is suggested to be used for selection of breeding germplasm [16]. This QTL was significantly associated with dry root weight (dry root biomass) under both control and drought stress conditions in spring wheat cultivars of Pakistan. The favorable haplotype allele frequency was higher in cultivars released in rainfed areas, however, some old cultivars like C-217 and C-518 also carried favored allele. Wheat LBD (LARGE ORGAN BOUNDARIES DOMAIN) gene *TaMOR* also plays a significant role in wheat root development and improvement of root systems [42]. Of the 12 genotypes tested, we found the highest RNA-seq based *TaMOR* expression in five wheat cultivars released in 1965 of which Zincol-2016, GA-2002, and Chakwal-50 belong to the post-2000 breeding group. In line with our results, Li et al. [43] reported that overexpression of *TaMOR* in rice plants contributed to the larger root system and higher grain yield in rice plants.

## Conclusion

It is concluded from our findings that the historical bread wheat cultivars released before the green revolution (1965) have poor root systems as compared to cultivars released thereafter. Cultivars of the post-1965 group have improved root systems under both well-watered and water-limited conditions and thus can tolerate climatic fluctuations. A significant improvement in drought-adaptive traits such as the depth and steep angle frequency was prominent feature of modern cultivars. The favorable allele of a major root biomass QTL, qDRM-5B, was present in low frequency, and its positive selection in breeding could improve drought adaptive RSA. This progressive improvement in RSA is also linked to green revolution *Rht* genes along with other phenology-related genes such as *TaLTPs, TaSus-2B, TaMOR*, *and DRO1* that have significant allelic effects on RSA traits. Furthermore, correlation analysis of root and yield-related indicates a strong association of root systems with grain yield. Thus, we suggest that it is crucial to integrate the knowledge of promising root systems and root traits into breeding programs to develop climate-resilient varieties.

## Supplementary Information

**Additional file 1: Table S1.** The raw data for RT-PCR experiment.

## Data Availability

The RNAseq data used in this experiment is available under NCBI BioProject ID PRJNA863398. All the other supporting data is available as supplementary files. The tpm values were extracted from RNAseq data and raw file is deposited at DryAd and can be accessed from: 10.5061/dryad.zs7h44jcs.
